# Real-World Evaluation of Modern Adjuvant Radiotherapy in Women with Stage IB Endometrial Cancer

**DOI:** 10.3390/cancers13061386

**Published:** 2021-03-18

**Authors:** Jenny Ling-Yu Chen, Chao-Yuan Huang, Yu-Sen Huang, Che-Yu Hsu, Keng-Hsueh Lan, I-Lun Shih, Wen-Fang Cheng, Chi-An Chen, Bor-Ching Sheu, Sung-Hsin Kuo

**Affiliations:** 1 Department of Radiology, National Taiwan University College of Medicine, Taipei 100, Taiwan; lychen@ntu.edu.tw (J.L.-Y.C.); yusenh@ntu.edu.tw (Y.-S.H.); rinkoss@ntuh.gov.tw (I.-L.S.); 2 Division of Radiation Oncology, Department of Oncology, National Taiwan University Hospital, Taipei 100, Taiwan; codiwa@ntuh.gov.tw (C.-Y.H.); khlan@ntuh.gov.tw (K.-H.L.); shkuo101@ntu.edu.tw (S.-H.K.); 3 Cancer Center, College of Medicine, National Taiwan University, Taipei 100, Taiwan; 4 Department of Medical Imaging and Radiological Technology, Yuanpei University, Hsinchu 300, Taiwan; 5 Department of Medical Imaging, National Taiwan University Hospital, Taipei 100, Taiwan; 6 Department of Obstetrics and Gynecology, National Taiwan University Hospital, Taipei 100, Taiwan; wenfangcheng@ntuh.gov.tw (W.-F.C.); chianchen@ntu.edu.tw (C.-A.C.); bcsheu@ntu.edu.tw (B.-C.S.)

**Keywords:** endometrioid adenocarcinoma, stage IB, modern radiotherapy, external beam radiotherapy, vaginal brachytherapy

## Abstract

**Simple Summary:**

Endometrial cancer is the most common cancers of the female genital tract. However, the optimal adjuvant treatment for stage IB endometrial cancer is not well-defined. We aimed to study the benefit of modern adjuvant radiotherapy for women with stage IB endometrial cancer. We found that adjuvant external-beam radiotherapy significantly improved survival in patients with stage IB endometrial cancer. However, the benefit of adjuvant radiotherapy varied among the patients, suggesting that the treatment should be individualized.

**Abstract:**

The optimal adjuvant treatment for stage IB endometrial cancer remains undefined. We investigated the benefit of modern adjuvant radiotherapy for women with stage IB endometrial cancer. We retrospectively reviewed patients with surgically staged, pure stage IB endometrioid adenocarcinoma (2010 to 2018). Adjuvant modern radiotherapy consists of external-beam radiotherapy (EBRT) by intensity, volumetric-modulated arc radiotherapy, or image-guided vaginal brachytherapy (VBT). The study included 180 stage IB patients. Patients with grade 3 diseases had frequent aggressive histology patterns (lymphovascular space invasion (LVSI); low uterine segment involvement) and experienced significantly shorter recurrence-free survival (RFS) and overall survival (OS) than patients with grade 1/2 diseases. Adjuvant modern radiotherapy decreased the incidence of acute/chronic grade ≥2 gastrointestinal toxicity. In IB grade 1/2 patients, EBRT significantly lengthened survival (RFS/OS); patients with age >60 years, myometrial invasion beyond the outer third, or LVSI benefited the most from EBRT. EBRT also significantly improved survival (RFS/OS) in IB grade 3 patients, where patients with bulky tumors or LVSI benefited the most from EBRT. Therefore, EBRT may be beneficial for all stage IB patients.

## 1. Introduction

Endometrial cancer is the most common malignancy of the female genital tract, with a continuously increasing incidence rate [1]. The 2009 International Federation of Gynecology and Obstetrics (FIGO) staging system for endometrial carcinoma defined stage IB as a disease involving the invasion of more than half of the myometrium, which comprises a wide spectrum of risk according to pathologic and age-based risk factors, and the optimal adjuvant management for these patients is not well-defined [2,3]. Several landmark clinical trials have attempted to identify women at risk for recurrence, so that the optimal treatment paradigm could be determined. Both the GOG-99 and PORTEC-1 studies, including stage IB patients, demonstrated benefits for adjuvant external beam radiotherapy (EBRT) on local control and disease-free survival, with further analysis confirming the beneficial necessity of EBRT in IB grade 3 patients [4,5]. Meanwhile, the PORTEC-2 study suggested that vaginal brachytherapy (VBT) alone may be the standard treatment option in IB grade 1/2 patients, with fewer toxic effects than the EBRT group at the cost of higher local recurrence (vaginal or pelvic) [6]. These findings indicate that in certain IB grade 1/2 patients with unfavorable risk factors, instead of receiving VBT alone, they may benefit more from EBRT.

Modern adjuvant radiotherapy for endometrial cancers consists of intensity-modulated radiation therapy (IMRT), volumetric modulated arc therapy (VMAT), and image-guided VBT, all of which demonstrate equivalent and even superior disease control with reduced treatment-related toxicity and are becoming more widely available [7,8,9,10]. Modern radiotherapy has the advantages of three-dimensional computed tomography (CT) simulation for organ motion management, Monte Carlo algorithm treatment planning for precise dose distribution, multileaf collimator integration, and cone-beam CT verification of patient position in linear accelerators; this helps to deliver precise radiation dosages to the target while minimizing the exposure to organs at risk, leading to increased locoregional tumor control, decreased radiation-related side effects, and better survival rates than conventional radiation delivery [11,12].

While there is a consensus regarding the need for EBRT in IB grade 3 patients, the guidelines from the National Comprehensive Cancer Network (NCCN) [13], American Society for Radiation Oncology (ASTRO) [14], and the European Society for Radiotherapy and Oncology (ESTRO) [15] permit substantial variability in the management of IB grade 1/2 patients, including VBT alone, EBRT, and observation, based on pathologic and age-based risk factors. In this study, we wish to investigate the merits of modern adjuvant radiotherapy for stage IB endometrial cancer.

## 2. Methods and Materials

### 2.1. Study Design and Patient Selection

The National Taiwan University Hospital Research Ethics Committee approved the study (approval number: 201910019RINA). Between 2010 and 2018, we retrospectively included endometrial cancer patients who received staging surgery at the National Taiwan University Hospital, the affiliated Yun-Lin branch, and the affiliated Hsin-Chu branch). We identified a total of 1273 patients with surgically staged endometrial cancer, of whom 203 had FIGO stage IB disease according to the 2009 International FIGO staging system for endometrial cancer [2]. We excluded patients who were nonendometrioid histology or mixed histology types, patients who received chemotherapy or radiotherapy before surgery, and patients who underwent palliative surgery. Finally, 180 surgically staged patients with stage IB pure endometrioid adenocarcinoma were ultimately included.

### 2.2. Surgery

For apparent uterine-confined endometrial carcinoma, the primary treatment was staging surgery, which included total hysterectomy, bilateral salpingo-oophorectomy, selective or systematic pelvic lymphadenectomy, and peritoneal washings [7,16]. The median dissected lymph nodes number was 16 (range: 0–43). Minimally invasive surgical techniques using a vaginal, laparoscopic, or robotic approach were considered according to the physician’s preferences. Pathologic evaluation of all tissue specimens was performed by gynecologic pathologists at our institution. “Low uterine segment involvement” was defined as when tumor involvement of the low uterine segment was documented by either histologic or gross pathologic description in the pathology report [17].

### 2.3. Chemotherapy

Chemotherapy was administered less frequently than radiotherapy in IB patients. Platinum-based regimens plus paclitaxel were commonly given (75%), while other regimens including cisplatin plus doxorubicin (13%), doxorubicin plus ifosfamide (6%), or epirubicin plus cisplatin (6%) were also administered according to the physician’s preferences [7]. The median number of administered chemotherapy cycles was four (range: 2–6). Adjuvant chemotherapy typically began within 6 weeks of surgery. Women who received both adjuvant chemotherapy and radiotherapy completed all chemotherapy courses before radiotherapy (69%) or in conjunction with radiotherapy in a sandwich pattern (31%).

### 2.4. Radiotherapy

**Figure 1:**
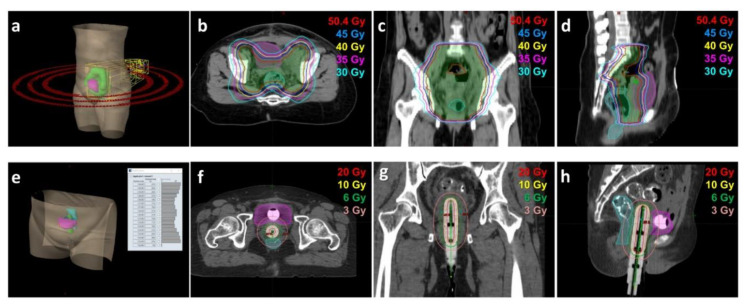
Modern radiotherapy techniques and dose distributions. Upper panels: isodose distributions in a patient with stage IB grade 3 endometrial cancer who underwent adjuvant radiotherapy via volumetric modulated arc therapy (VMAT). A 50.4-Gy dose (28 fractions) was prescribed to target volumes. (**a**) Beam arrangement according to VMAT plan. Dose distributions in the axial (**b**), coronal (**c**), and sagittal (**d**) views. Green, pink, and cyan areas indicate target volumes, bladder, and rectum, respectively. Red, blue, yellow, pink, and indigo lines represent isodose curves of 50.4, 45, 40, 35, and 30 Gy, respectively. Lower panels: isodose distributions in a patient with stage IB grade-1 endometrial cancer who underwent adjuvant image-guided high-dose-rate vaginal brachytherapy. A 30-Gy dose (3 fractions) was prescribed to the vaginal stump and the upper two-thirds of the mucosa. (**e**) Brachytherapy dose delivered using the “Nucletron HDR” ^192^Ir remote afterloading technique. Dose distributions in the axial (**f**), coronal (**g**), and sagittal (**h**) views. Green, pink, and cyan areas indicate target volumes, bladder, and rectum, respectively. Red, yellow, green, and coral lines represent isodose curves of 20, 10, 6, and 3 Gy, respectively.

For stage IB endometrial cancer, adjuvant radiotherapy is comprised of VBT, EBRT, or both [13]. Modern radiotherapy including IMRT and VMAT has been adopted by our healthcare system since 2010, in order to minimize the dosage given to normal organs on the basis of adjuvant radiotherapy [11,18]. EBRT dose was 50.4 Gy in 28 fractions over six weeks, targeting the lower common iliacs, external iliacs, internal iliacs, obturators, parametria, upper vagina and paravaginal tissue, as per the updated delineation consensus for gynecologic malignancy [7,11,19]. The Varian TrueBeam™ Radiotherapy System (Varian, Palo Alto, CA, USA) or the Elekta Synergy accelerator (Elekta, Stockholm, Sweden) administered EBRT with multiple coplanar ports [20]. In VBT, high dose-rate brachytherapy via a vaginal cylinder was used to irradiate the vagina cuff using the “Nucletron HDR” ^192^Ir remote afterloading technique [21,22]. Patients receiving VBT only had brachytherapy doses of 10 Gy per fraction for 3 fractions prescribed to the vaginal mucosa, and those receiving VBT boost after EBRT had brachytherapy doses of 6 Gy per fraction for 2 fractions prescribed to the vaginal mucosa. Examples of modern radiotherapy techniques, including EBRT and VBT with associated isodose curves, are shown in Figure 1.

### 2.5. Statistical Analysis

Statistical analyses were performed using the Statistical Package for Social Sciences for Windows, version 17.0 (SPSS, Chicago, IL, USA). Distribution differences of clinical and pathological characteristics among patients with grade 1/2 or grade 3 disease were analyzed using Fisher’s exact test, and mean values were compared using Student’s *t*-test. The Cancer Registry Medical Information Management Office in the healthcare system provided the survival data available on June 30, 2020. All patients were followed every 3–6 months as routine clinical practice until recurrence or death [16,22]. Recurrent tumors in the pelvis, or pelvic/para-aortic lymphadenopathy were defined as locoregional recurrence. Failures outside the locoregional area detected by pathology, cytology, or radiology were defined as distant metastasis. All events were calculated from the date of treatment completion. Kaplan–Meier life-table analyses and log-rank tests were used to assess recurrence-free survival (RFS) and overall survival (OS) rates and to classify cases according to prognostic parameters. Prognostic variables found to be significant in univariate analysis were included in the multivariate analysis using a Cox proportional hazards regression model. A *p*-value of ≤0.05 was considered statistically significant.

## 3. Results

### 3.1. Patient Characteristics

A total of 180 patients with stage IB endometrioid carcinoma were identified, and their characteristics are listed in Table 1. Overall, the median age was 61.9 years, and one-fourth of patients underwent minimally invasive surgery, including vaginal, robotic-assisted, or laparoscopic surgery. The mean tumor size was 4.2 cm, the depth ratio of myometrial invasion was 70%, and nearly one-tenth (9%) had low uterine segment involvement. Lymphovascular space invasion (LVSI) was present in nearly half (45%) of the tumors, and abnormal peritoneal washing cytology was found in 13% of the total patients. Nearly one-fourth (24%) of the patients presented with positive peritoneal washing cytology. Of these, 41 women had IB grade 3 diseases. When comparing grade 3 to grade 1/2 patients, more patients in the grade 3 group presented with aggressive histology patterns, including low uterine segment involvement (17% in grade-3 group vs. 7% in grade 1/2 group, *p* = 0.046) and LVSI (59% in grade 3 group vs. 41% in grade 1/2 group, *p* = 0.042).

### 3.2. Adjuvant Treatments and Outcomes

With respect to adjuvant therapy, adjuvant radiotherapy was more frequently suggested for stage IB patients (58%), and among the 104 patients receiving adjuvant radiotherapy, VBT alone was administered for 49% of patients and EBRT ± VBT boost was administered for 51% of patients. In the 53 patients who received adjuvant EBRT ± VBT boost, IMRT was used for 42% of patients, and VMAT was used for 58% of the patients. The majority (46 of 53 patients, 87%) of the patients who received adjuvant radiotherapy had both EBRT and VBT. Additionally, 9% received chemotherapy, and 37% did not receive any kind of adjuvant treatment.

Of the eight IB grade 3 patients who did not receive any kind of adjuvant treatments, four decided against adjuvant therapy, and four had comorbidities that precluded such treatments. As shown in Table 1, there were significantly more grade 3 patients who received adjuvant chemotherapy (*p* = 0.012) and adjuvant EBRT ± VBT boost (*p* < 0.001) compared to patients with grade 1/2 diseases, indicating the necessity of adjuvant treatments in grade 3 patients.

Regarding gastrointestinal side effects by modern adjuvant radiotherapy, one fifth of patients (20%) experienced acute grade 2 diarrhea, and few (1%) experienced acute grade 3 diarrhea; few (4%) had late grade 2 diarrhea requiring medications, one patient experienced late grade 3 radiation proctitis treated by colonoscopy argon plasma coagulation, and one patient experienced late grade 4 gastrointestinal toxicity of bowel abscess and fibrosis managed by surgical management (right hemicolectomy and anterior resection with adhesiolysis).

**Figure 2:**
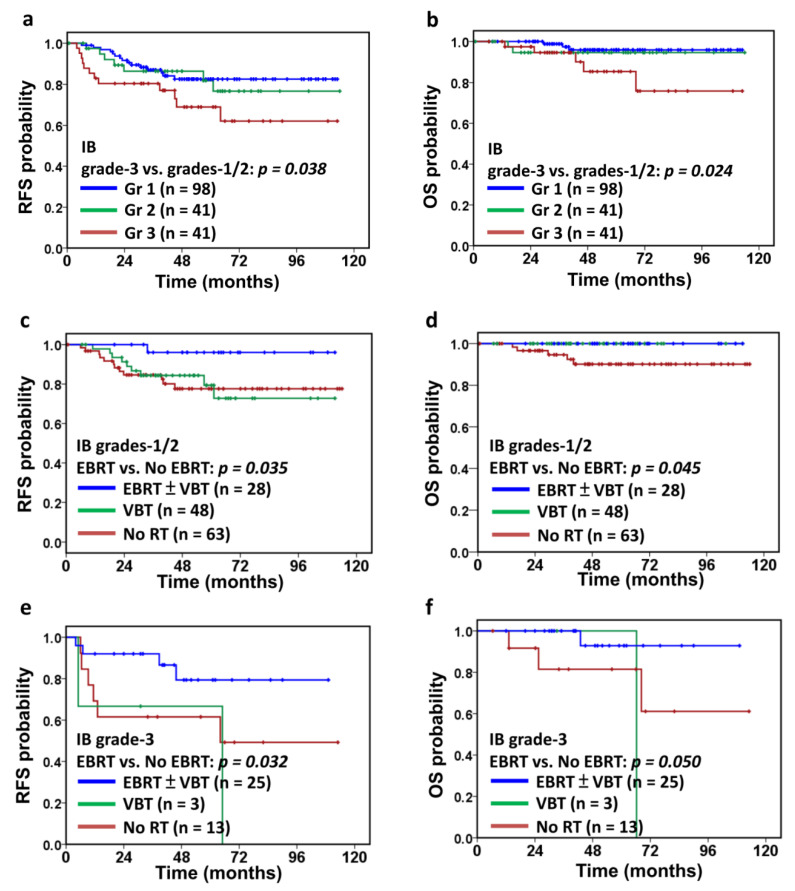
Survival in patients with stage IB endometrial cancer by pathology grade and by type of adjuvant radiotherapy. Recurrence-free survival (RFS) (**a**) and overall survival (OS) (**b**) of patients with stage IB endometrial cancer based on the pathology grade. Recurrence-free survival (RFS) (**c**) and overall survival (OS) (**d**) of stage IB grades-1/2 patients according to adjuvant radiotherapy: external beam radiotherapy (EBRT) ± vaginal brachytherapy (VBT) boost, VBT alone, or no radiotherapy. RFS (**e**) and OS (**f**) of stage IB grade-3 patients according to adjuvant radiotherapy. *p*-values were determined using the Kaplan–Meier log-rank test.

The median follow-up was 50.9 months (range, 2.0–114.0 months). There were 34 patients with tumor recurrence, with locoregional recurrence in 20 (pelvic or para-aortic recurrence, or vaginal stump recurrence) and distant metastasis in 21 (liver, bone, lung, brain, peritoneal carcinomatosis, or distant lymphadenopathy). Nine patients had both distant metastasis and locoregional recurrence. A total of ten patients (5%) died; most deaths (90%) were attributed to cancer progression. Patients with IB grade 3 disease experienced significantly shorter RFS (*p* = 0.03, Figure 2a) and OS (*p* = 0.024, Figure 2b) when compared to grade 1/2 patients.

### 3.3. Parameters Affecting Survivals in IB Patients

As shown in Table 2, older age, LVSI, and positive peritoneal washing cytology in IB grade 1/2 patients were associated with a trend toward increased risk of tumor recurrence (hazard ratio (HR) of age > 60 years: 2.4, *p* = 0.074; HR of LVSI: 2.5, *p* = 0.076; HR of positive peritoneal washing cytology: 2.1, *p* = 0.042) and significant risks of death (HR of age > 60 years: 4.8, *p* = 0.040; HR of LVSI: 4.2, *p* = 0.048; HR of positive peritoneal washing cytology: 4.4, *p* = 0.046). While the administration of adjuvant chemotherapy neither did decrease the risk of tumor recurrence nor improve survival, the administration of adjuvant EBRT ± VBT boost was associated with a significantly longer RFS (*p* = 0.035, Figure 2c) and OS (*p* = 0.045, Figure 2d). After excluding patients who received chemotherapy, the administration of adjuvant EBRT ± VBT boost persistently decreased tumor recurrence in IB grade 1/2 patients (*n* = 131, *p* = 0.046). 

As shown in Table 3, positive peritoneal washing cytology was likewise associated with significantly increased risks of tumor recurrence in IB grade 3 patients (HR: 5.9, *p* = 0.004) and a trend toward death (HR: 4.3, *p* = 0.171). Once again, the administration of adjuvant EBRT ± VBT boost was associated with longer RFS (*p* = 0.032, Figure 2e) and a trend toward longer OS (*p* = 0.050, Figure 2f). Moreover, after excluding patients who received chemotherapy, the administration of adjuvant EBRT ± VBT boost remained significant in preventing tumor recurrence (*n* = 33, *p* = 0.047).

### 3.4. Effectiveness of Adjuvant External-Beam Radiotherapy

**Figure 3:**
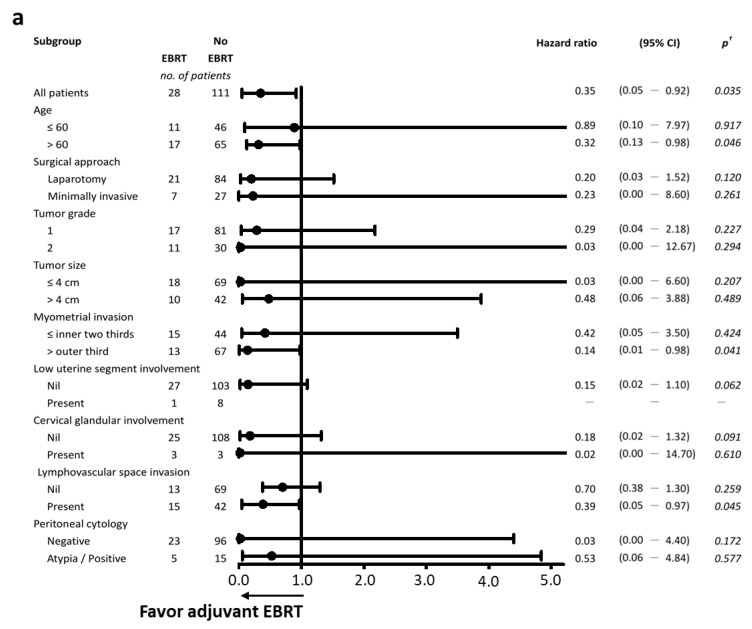

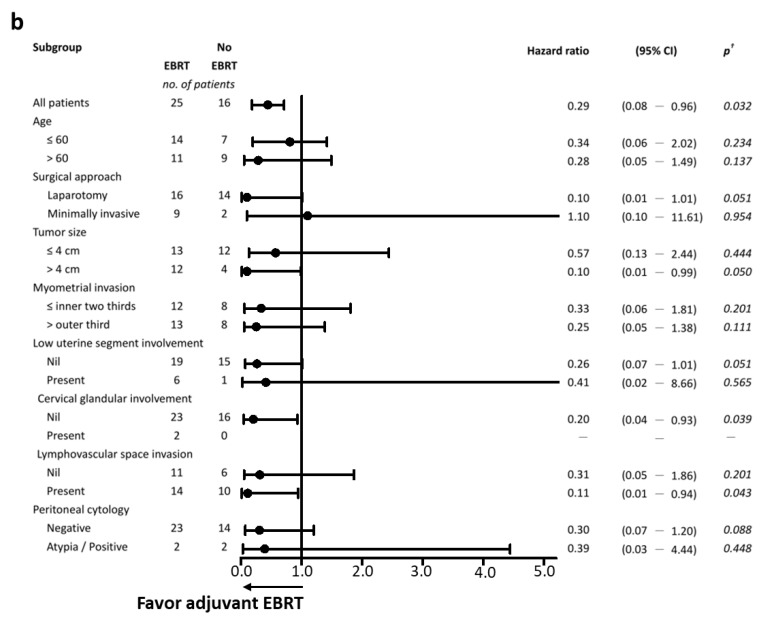
Subgroup analysis of prognostic factors for recurrence-free survival in patients with stage IB endometrial cancer by pathology grade. (**a**) Grades 1/2 (*n* = 139); (**b**) grade 3 (*n* = 41). Hazard ratios and 95% confidence intervals (CI) were calculated using Cox proportional hazards regression. Minimally invasive surgery included vaginal, robotic-assisted, or laparoscopic surgery. EBRT, external-beam radiotherapy. ^†^ Significance was tested using Kaplan–Meier life table analysis and the log-rank test.

As shown in Figure 3, adjuvant EBRT was beneficial to both IB patients with grade 1/2 disease (HR: 0.35, *p* = 0.035) and IB patients with grade 3 disease (HR: 0.29, *p* = 0.032), leading to significantly longer RFS; after multivariate analysis, adjuvant EBRT remained a significant factor in preventing recurrence (Table 4). In IB grade 1/2 patients, identifying patients who might benefit the most from adjuvant EBRT was determined by subgroup analysis (Figure 3a). Adjuvant EBRT decreased tumor recurrence risk among older patients (*p* = 0.046), as well as those with myometrial invasion beyond the outer third (*p* = 0.041) and LVSI (*p* = 0.045). In IB grade 3 patients, adjuvant EBRT decreased the risk of tumor recurrence among patients with bulky tumors (>4 cm, *p* = 0.050) and LVSI (*p* = 0.043) (Figure 3b).

## 4. Discussion

Our study emphasizes the value of modern adjuvant radiotherapy, including EBRT by IMRT or VMAT or image-guided VBT, on clinical survival, specifically in women with surgically staged IB endometrial cancer. We found that IB grade 3 patients had significantly worse survival outcomes than those with grade 1/2 diseases. Furthermore, modern adjuvant EBRT may be helpful for all stage IB patients, with confirmed benefits in IB grade 3 patients, as well as those with IB grade 1/2 patients with unfavorable risk factors, including old age, deep myometrial invasion, and LVSI.

Patients with surgically staged IB diseases represent a heterogeneous population [23,24,25]. Landmark clinical trials have confirmed the beneficial necessity of EBRT in IB grade 3 patients but showed variability in IB grade 1/2 patients. The GOG-99 and PORTEC-1 studies demonstrated that EBRT could reduce recurrence in stage IB patients with at least two unfavorable risk factors, including increasing age, grade ≥ 2, LVSI, or outer-third myometrial invasion [4,5]. Moreover, the ASTEC EN.5 trial and a Norwegian trial demonstrated confirmed EBRT benefits on reducing vaginal or pelvic recurrence; however, they were accompanied by acute and late toxicities, thus discouraging the use of EBRT in IB patients [26,27]. With the technical improvement of modern radiotherapy, giving precise radiation dosages to the target while minimizing the exposure to organs at risk and therefore decreasing radiation-related side effects may further promote the use of EBRT in adjuvant treatments, as shown in our results.

The benefit of adjuvant radiotherapy varied among the stage IB cohort, suggesting that treatment should be individualized [28,29]. The PORTEC2 study demonstrated that patients receiving EBRT experienced acute and late bowel symptoms with worse social functioning, thus suggesting VBT alone as an appropriate option in IB grade 1/2 patients, but at the cost of higher local recurrence (vaginal or pelvic) [6,30]. Locoregional recurrence may place patients at high risk for synchronous or metachronous regional or distant failures, which have poor outcomes. Improved local control from adjuvant radiation may translate into improved overall survival as demonstrated by our results and others, suggesting that EBRT should be considered for IB grade 1/2 patients with unfavorable risk factors.

In the present study, a similar magnitude of RFS was observed between IB grade 1/2 patients receiving VBT vs. no RT (Figure 2c and Table 2). According to the 10-year results of the PORTEC-2 trial [30], patients receiving VBT had a 5-year RFS of 81% and a 10-year RFS of 67%, which is compatible with our data where IB grade 1/2 patients receiving VBT had a 5-year RFS of 79% and a 9-year RFS of 73%. Due to the retrospective study design, unfavorable risk factors may not be balanced between groups; patients with unfavorable risk factors for adjuvant radiotherapy but with comorbidities precluding EBRT might be assigned to the VBT group. In IB grade 1/2 patients with unfavorable risk factors, adjuvant VBT may be relatively insufficient and adjuvant EBRT should be considered to decrease tumor recurrence. 

Minimally invasive surgery has established advantages and is considered the surgical treatment option for endometrial carcinoma patients [31]. In the present study, we did not find a significant survival disparity between different surgical approaches (laparotomy, *n* = 135 vs. minimally invasive, *n* = 45). After a post hoc analysis, the sample size of 180 patients only provides 69% power to detect a difference in the mean RFS of 93 ± 4 months in patients who underwent laparotomy surgery compared to 95 ± 6 months, with an α level of 0.05. Therefore, due to inadequate power, the influence of different surgical approaches on survival should be interpreted carefully and may need further investigation in prospective clinical trials.

LVSI refers to the presence of cancer cells within the lymphatic or vascular space of the uterus and is confirmed as a risk factor for distant metastasis, pelvic recurrence, and shorter overall survival in prospective studies. Therefore, some authors advocate systemic therapy in this subgroup of patients [32]. In our results, LVSI was present in nearly half of the stage IB patients and presented more frequently in grade 3 patients, and adjuvant EBRT had beneficial results in decreasing cancer recurrence in both IB grade 3 and grade 1/2 patients who presented with LVSI pathology characteristics. Prospective studies (PORTEC-3 and GOG-249) examining whether adjuvant EBRT is adequate or if combined and adjuvant systemic therapy are needed have also shown beneficial results when using adjuvant EBRT alone in this subgroup of patients. These studies prove that pelvic RT alone remains an effective, well-tolerated, and appropriate adjuvant treatment in stage IB patients with LVSI [33,34].

The strength of our study includes its basis in multi institutional, real-world patterns of care and outcome, and the proven overall survival benefits. The main limitation of this study is its retrospective design, which may be subject to confounding factors. The patient population was heterogeneous in terms of grade, characteristics, and adjuvant treatments with multimodality. Further, longer follow-up times may be required to further investigate patient outcomes during future research trials.

## 5. Conclusions

Our data support the use of adjuvant modern radiotherapy to improve survival in women with surgically staged IB pure endometrioid adenocarcinoma, despite the abovementioned limitations. In patients with IB grade 3 disease, adjuvant modern EBRT resulted in confirmed survival advantages. In IB grade 1/2 patients with unfavorable risk factors (e.g., including old age, deep myometrial invasion, and LVSI), adjuvant modern EBRT conferred additional survival benefits and may be considered to decrease tumor recurrence. These findings should be further investigated in prospective clinical trials.

## Figures and Tables

**Table 1:** Patients’ demographics and tumor characteristics (*n* = 180).

Characteristic No. of Patients (%)	All (*n* = 180)	Grade 1–2 (*n* = 139)	Grade 3 (*n* = 41)	*p-Value*
Age (years), mean (range)	61.9 (35.5–86.6)	62.1 (37.0–86.6)	61.3 (35.5–83.1)	0.673 *
Surgical approach				0.838 ^†^
Laparotomy	135 (75%)	105 (76%)	30 (73%)	
Minimally invasive *	45 (25%)	34 (24%)	11 (27%)	
Tumor size (cm)				0.669 *
Mean (range)	4.2 (1.0–14.0)	4.1 (1.0–14.0)	4.3 (1.0–10.0)	
Myometrial invasion				0.468 *
Mean (range)	0.70 (0.50–1.00)	0.70 (0.50–1.00)	0.72 (0.50–1.00)	
Low uterine segment involvement				0.046 ^†^
Nil	164 (91%)	130 (94%)	34 (83%)	
Present	16 (9%)	9 (6%)	7 (17%)	
Cervical glandular involvement				1.000 ^†^
Nil	172 (96%)	133 (96%)	39 (95%)	
Present	8 (4%)	6 (4%)	2 (5%)	
Lymphovascular space invasion				0.042 ^†^
Nil	99 (55%)	82 (59%)	17 (41%)	
Present	81 (45%)	57 (41%)	24 (59%)	
Peritoneal washing cytology				0.603 ^†^
Negative	156 (87%)	119 (86%)	37 (90%)	
Atypia/Positive	24 (13%)	20 (14%)	4 (10%)	
Adjuvant chemotherapy				0.012 ^†^
No	164 (91%)	131 (94%)	33 (80%)	
Yes	16 (9%)	8 (6%)	8 (20%)	
Adjuvant radiotherapy				<0.001 ^†^
No	76 (42%)	63 (45%)	13 (32%)	
VBT	51 (28%)	48 (35%)	3 (7%)	
EBRT ± VBT boost	53 (30%)	28 (20%)	25 (61%)	

Abbreviations: VBT = vaginal brachytherapy; EBRT = external-beam radiotherapy * Minimally invasive surgery including vaginal, robotic-assisted, or laparoscopic surgery ^†^ Significance tested using Fisher’s exact test. * Significance tested using Student’s t test.

**Table 2:** Parameter analysis of potential prognostic factors in IB grade 1–2 patients.

	5-Year RFS	HR (95% CI)	*p*-Value ^†^	5-Year OS	HR (95% CI)	*p*-Value ^†^
Age (years)			0.074			0.040
≤60	89	—		100	—	
>60	78	2.4 (0.9–6.5)		93	4.8 (1.1–7.5)	
Surgical approach			0.415			0.736
Laparotomy	81	—		96	—	
Minimally invasive *	87	0.6 (0.2–1.9)		96	0.7 (0.1–6.2)	
Tumor size			0.120			0.183
≤4 cm	83	—		100	—	
>4 cm	81	1.2 (0.4–2.5)		93	3.0 (0.0–19.3)	
Myometrial invasion			0.084			0.333
≤Inner two thirds	84	—		94	—	
>Outer third	78	1.5 (0.6–3.6)		97	2.8 (0.3–15.3)	
Low uterine segment involvement			0.076			0.552
Nil	84	—		100	—	
Present	78	2.5 (1.0–5.0)		95	2.0 (0.0–16.7)	
Cervical glandular involvement			0.946			0.593
Nil	83	—		95	—	
Present	82	1.1 (0.1–8.0)		100	2.2 (0.0–7.5)	
Lymphovascular space invasion			0.076			0.048
Nil	84	—		92	—	
Present	78	2.5 (1.0–5.0)		100	4.2 (1.0–14.5)	
Peritoneal cytology			0.042			0.046
Negative	84	—		97	—	
Atypia/Positive	64	2.1 (1.0–5.6)		90	4.4 (1.1–16.5)	
Adjuvant chemotherapy			0.611			0.575
No	82.6	—		95	—	
Yes	75.0	1.5 (0.3–6.2)		100	0.1 (0.0–8.1)	
Adjuvant radiotherapy			0.048			0.040
No	78	—		90	—	
VBT	79	0.9 (0.4–2.3)		100	0.4 (0.0–8.2)	
EBRT ± VBT boost	96	0.3 (0.0–0.9)		100	0.3 (0.0–9.3)	

Abbreviations: VBT, vaginal brachytherapy; EBRT, external-beam radiotherapy; RFS, recurrence-free survival; OS, overall survival; HR, hazard ratio; CI, confidence interval * Minimally invasive surgery including vaginal, robotic-assisted, or laparoscopic surgery ^†^ Significance was tested using Kaplan–Meier life table analysis and the log-rank test.

**Table 3:** Parameter analysis of potential prognostic factors in IB grade 3 patients.

	5-Year RFS	HR (95% CI)	*p*-Value ^†^	5-Year OS	HR (95% CI)	*p*-Value ^†^
Age (years)			0.380			0.445
≤60	70	—		86	—	
>60	68	1.7 (0.5–5.3)		84	1.9 (0.3–12.1)	
Surgical approach			0.600			0.675
Laparotomy	70	—		79	—	
Minimally invasive *	68	1.4 (0.4–4.6)		100	1.6 (0.2–14.4)	
Tumor size			0.557			0.979
≤4 cm	72	—		87	—	
>4 cm	67	1.4 (0.4–4.8)		83	1.0 (0.2–5.9)	
Myometrial invasion			0.913			0.316
≤Inner two thirds	73	—		89	—	
>Outer third	63	1.1 (0.3–3.4)		82	2.9 (0.3–27.0)	
Low uterine segment involvement			0.820			0.783
Nil	69	—		83	—	
Present	86	1.2 (0.3–5.6)		50	1.4 (0.1–12.5)	
Cervical glandular involvement			0.421			0.637
Nil	72	—		85	—	
Present	50	2.3 (0.3–18.1)		100	0.1 (0.0–37.3)	
Lymphovascular space invasion			0.666			0.418
Nil	75	—		81	—	
Present	65	1.3 (0.4–4.3)		94	2.4 (0.3–21.6)	
Peritoneal cytology			0.004			0.171
Negative	76	—		87	—	
Atypia/Positive	25	5.9 (1.5–23.5)		67	4.3 (0.5–24.8)	
Adjuvant chemotherapy			0.387			0.114
No	76	—		84	—	
Yes	50	1.8 (0.5–6.8)		50	3.9 (0.8–14.7)	
Adjuvant radiotherapy			0.039			0.097
No	62	—		82	—	
VBT	67	2.6 (0.5–13.6)		100	2.6 (0.7–10.3)	
EBRT ± VBT boost	79	0.4 (0.1–1.5)		93	0.3 (0.1–2.9)	

Abbreviations: VBT, vaginal brachytherapy; EBRT, external-beam radiotherapy; RFS, recurrence-free survival; OS, overall survival; HR, hazard ratio; CI, confidence interval * Minimally invasive surgery including vaginal, robotic-assisted, or laparoscopic surgery ^†^ Significance was tested using Kaplan–Meier life table analysis and the log-rank test.

**Table 4:** Multivariate analysis of prognostic factors on recurrence-free survival in IB patients.

	IB Grade 1–2		IB Grade 3	
	HR (95% CI)	*p*-Value *	HR (95% CI)	*p*-Value *
Peritoneal cytology Atypia/Positive vs. Negative	2.3 (0.8–6.2)	0.110	6.5 (1.6–26.5)	0.010
Adjuvant radiotherapy EBRT ± VBT boost vs. No/VBT	0.1 (0.0–1.0)	0.049	0.3 (0.1–0.9)	0.033

Abbreviations: VBT, vaginal brachytherapy; EBRT, external-beam radiotherapy; HR, hazard ratio; CI, confidence interval. * Significance tested using the Cox proportional hazards regression model.

## Data Availability

All data analyzed during this study are included in this published article.
